# Technical Pitfalls for Fenestrated-Branched Endovascular Aortic Repair Following PETTICOAT

**DOI:** 10.1177/15266028231163439

**Published:** 2023-03-30

**Authors:** Aidin Baghbani-Oskouei, Emanuel R. Tenorio, Marina Dias-Neto, Andrea Vacirca, Aleem K. Mirza, Naveed Saqib, Bernardo C. Mendes, Laura Ocasio, Thanila A. Macedo, Gustavo S. Oderich

**Affiliations:** 1 Advanced Aortic Research Program, Department of Cardiothoracic and Vascular Surgery, McGovern Medical School, University of Texas Health Science Center at Houston, Houston, TX, USA; 2 Division of Vascular and Endovascular Surgery, Mayo Clinic, Rochester, MN, USA; 3 Department of Diagnostic and Interventional Imaging, McGovern Medical School, University of Texas Health Science Center at Houston, Houston, TX, USA

**Keywords:** aortic dissection, thoracoabdominal aneurysm, dissection stent, PETTICOAT, endovascular, FB-EVAR

## Abstract

**Purpose:**

The Provisional Extension to Induce Complete Attachment Technique (PETTICOAT) uses a bare-metal stent to scaffold the true lumen in patients with acute or subacute aortic dissections. While it is designed to facilitate remodeling, some patients with chronic post-dissection thoracoabdominal aortic aneurysms (TAAAs) require repair. This study describes the technical pitfalls of fenestrated-branched endovascular aortic repair (FB-EVAR) in patients who underwent prior PETTICOAT repair.

**Technique:**

We report 3 patients with extent II TAAAs who had prior bare-metal dissection stents treated by FB-EVAR. Two patients required maneuvers to reroute the aortic guidewire, which was initially placed in-between stent struts. This was recognized before the deployment of the fenestrated-branched device. A third patient had difficult advancement of the celiac bridging stent due to a conflict of the tip of the stent delivery system into one of the stent struts, requiring to redo catheterization and pre-stenting with a balloon-expandable stent. There were no mortalities and target-related events after a follow-up of 12 to 27 months.

**Conclusion:**

FB-EVAR following the PETTICOAT is infrequent, but technical difficulties should be recognized to prevent complications from the inadvertent deployment of the fenestrated-branched stent-graft component in-between stent struts.

**Clinical Impact:**

The present study highlights a few maneuvers to prevent or overcome possible complications during endovascular repair of chronic post-dissection thoracoabdominal aortic aneurysm following PETTICOAT. The main problem to be recognized is the placement of the aortic wire beyond one of the struts of the existing bare-metal stent. Moreover, encroachment of catheters or the bridging stent delivery system into the stent struts may potentially cause difficulties.

## Introduction

Thoracic endovascular aortic repair (TEVAR) has been widely used as the first line of treatment for complicated type B aortic dissections (TBADs) and has also gained acceptance in select patients with uncomplicated dissections who experience rapid aortic enlargement or have severe collapse of the true lumen.^1^ The strategy of TEVAR with the placement of distal bare-metal aortic stents to expand the true lumen (Provisional Extension To Induce Complete Attachment Technique [PETTICOAT]) is particularly useful in patients who present with renal and mesenteric malperfusion, have incomplete true lumen expansion following coverage of the primary entry tear or who develop a stent-induced new entry tear (SINE). In the chronic setting, PETTICOAT has not been shown to affect aortic remodeling and in fact, may create additional technical difficulties in patients who progress to develop chronic post-dissection thoracoabdominal aortic aneurysms (TAAAs) requiring additional repair.^2^

Fenestrated-branched endovascular aortic repair (FB-EVAR) has been widely used to treat patients with chronic post-dissection TAAAs with high technical success and relatively low mortality and morbidity.^3^ There is a paucity of reports on the use of FB-EVAR in patients who underwent prior placement of bare-metal stents. This study aimed to review the feasibility, technical pitfalls, and outcomes of FB-EVAR in patients with chronic post-dissection TAAAs who underwent prior PETTICOAT repair.

## Technique

We reviewed the clinical data of 686 consecutive patients treated by FB-EVAR for complex abdominal and TAAAs between 2007 and 2022 in 2 academic institutions by a single operating team. The study was approved by the institutional review board of both institutions with a data-sharing agreement, and all patients consented to participate in minimal-risk research studies. We found 50 patients (7.3%) who were treated for chronic post-dissection extent I to III TAAAs. There were 3 patients (0.4%) with extent II TAAAs and a mean age of 69±14 years old who had prior PETTICOAT procedures. The most indication for the PETTICOAT in all 3 cases was incomplete true lumen expansion following primary entry tear coverage as well as prevention of acute SINE, which developed immediately in 1 case after placement of the thoracic stent-graft and resulted in pressurization of the false lumen with the collapse of the true lumen distal to the edge of the stent-graft. The patients were treated with manufactured patient-specific or off-the-shelf fenestrated-branched stent-grafts by being enrolled in an ongoing prospective non-randomized physician-sponsored investigational device exemption (PS-IDE) study. The details of the 3 patients are summarized below:

### Case 1

**Figure 1.**
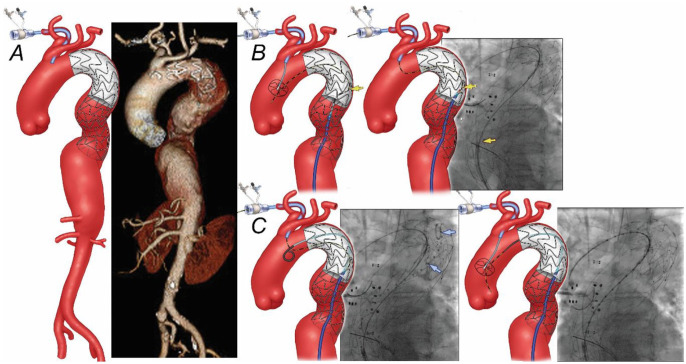
(A) Preoperative 3-dimensional computed tomography angiography reconstruction shows a 6.2 cm extent II thoracoabdominal aortic aneurysm distal to previous thoracic stent-graft and bare-metal dissection stent. (B) Snaring of a brachial-to-femoral guidewire was performed using a Kumpe catheter, which unfortunately was routed beneath one of the stent struts of the dissection stent (*yellow arrow*). (C) Once this issue was recognized, a “buddy” pigtail catheter was advanced proximally into the stent-graft (*blue arrow*), followed by redo snaring in the ascending aorta.

**Figure 2.**
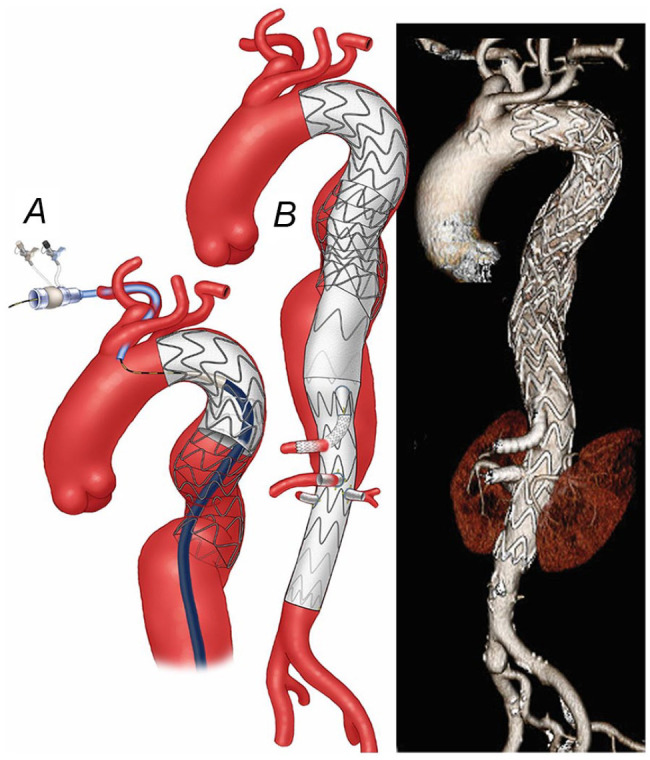
(A) Once the wire was confirmed to be in the central lumen of the stent, the fenestrated-branched stent-graft with a directional branch for the celiac axis and 3 fenestrations for the superior mesenteric artery and renal arteries was successfully deployed. (B) Postoperative 3-dimensional computed tomography angiography reconstruction shows the endovascular repair of an extent II thoracoabdominal aortic aneurysm with no kink, stent compressions, and endoleak.

A 76-year-old male patient presented with 6.2 cm extent II TAAA. The patient had been treated 7 months before by TEVAR and PETTICOAT using Zenith Dissection Endovascular System (Cook Medical, Bloomington, IN) for his subacute non-complicated TBAD (Figure 1). His medical history was notable for hypertension and hyperlipidemia. A FB-EVAR was planned using a patient-specific fenestrated-branched stent-graft with a 1-directional branch for the celiac axis (CA) and 3 fenestrations for the superior mesenteric artery (SMA) and bilateral renal arteries using a pre-loaded brachial-femoral delivery system. Following total percutaneous femoral access using pre-closure technique and surgical exposure of the right brachial artery, a 12 Fr Dry-Seal sheath (W. L. Gore & Associates, Inc., Flagstaff, AZ) was advanced via the brachial access into the thoracic stent-graft. Careful advancement of the femoral wire was performed using a pigtail catheter (Cook Medical, Bloomington, IN) to prevent engaging one of the struts of the bare-metal stent. Catheterization of the thoracic stent required to exchange to a Kumpe Access Catheter (Cook Medical, Bloomington, IN), and the wire was snared from the brachial approach. However, intravascular ultrasound and advancement of a 20 Fr 65 cm femoral sheath confirmed that the wire was placed beneath one of the stent struts in the overlapping area of the bare-metal stent and thoracic stent-graft (Figure 1). To overcome this, a “buddy” pigtail catheter was advanced via the 65 cm sheath and advanced forward into the stent-graft, allowing successful redo snaring from the brachial access. Once this was done and confirmed to be within the central lumen of the bare-metal stent, the fenestrated-branched stent-graft was successfully introduced via the brachial-femoral wire and deployed using a staggering technique with sequential catheterization and stenting of the target vessels with no endoleak, dissection, or embolization (Figure 2). The routine final rotational digital subtraction angiography (DSA) and cone-beam computed tomography (CBCT) showed widely patent CA, SMA, bilateral renal arteries, main body, and iliac limbs with no evidence of type I or III endoleak. The patient was dismissed on postoperative day 4 without complications. Follow-up computed tomography angiography (CTA) at 27 months has shown widely patent fenestrated-branched stent-graft with no endoleak and a 12 mm decrease in the aneurysm sac diameter.

### Case 2

**Figure 3.**
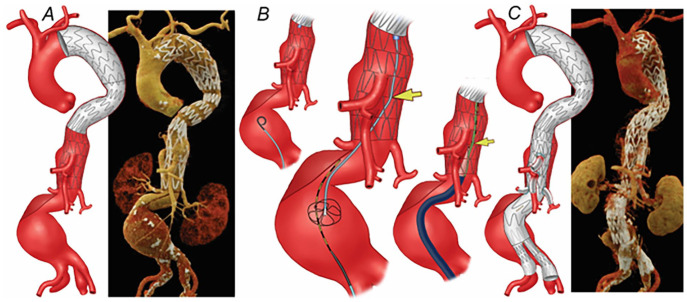
(A) Preoperative 3-dimensional computed tomography angiography (CTA) reconstruction shows a 6.8 cm extent II thoracoabdominal aortic aneurysm. A 4-vessel patient-specific fenestrated-branched stent-graft was designed for the patient. (B) During catheterization of the thoracic stent, the wire was noticed to be placed beneath one of the stent struts of the bare-metal stent, which prevented the introduction of the main graft. The same technique as described in Figure 1 was used for the catheterization of thoracic bare-metal stent. (C) Postoperative 3-dimensional CTA reconstruction showing the endovascular repair of the thoracoabdominal aortic aneurysm with no kink, stent compression, and endoleak.

A 73-year-old female patient was treated with TEVAR and PETTICOAT 3 weeks after experiencing TBAD. The patient’s medical history was notable for hypertension, morbid obesity, and diabetes mellitus. One month later, the patient presented with symptomatic, enlarging 6.8 cm extent II TAAA, and a 4-vessel patient-specific fenestrated-branched stent-graft was planned with a pre-loaded guidewire system. Total bilateral percutaneous femoral approach and right brachial approach were used. A 12 Fr Dry-Seal sheath was advanced from the right brachial artery to the descending thoracic aorta over an Amplatz Super Stiff guidewire (Boston Scientific, Natick, MA). A pigtail catheter was advanced via the right femoral approach across the bare-metal aortic stents and used for the advancement of a 0.035 inch Metro guidewire (Cook Medical, Bloomington, IN), which was snared from the right brachial access. True lumen access was confirmed with intravascular ultrasound. A 5 Fr 110 cm sheath was advanced over the metro wire to allow the placement of a second brachial-femoral wire. The 5 Fr sheath was withdrawn and readvanced via the second wire. The device and the 4 pre-loaded wires were advanced via the through-and-through wire and the 5 Fr sheath, respectively. However, despite careful advancement of the initial wire, this was inadvertently placed under one of the stent struts of the bare-metal stent within the thoracic aorta, which prevented the advancement of the fenestrated-branched stent-graft (Figure 3). Once the problem was recognized, the same maneuver of patient 1 was performed, and redo snaring was performed successfully. The fenestrated-branched stent-graft with pre-loaded wires was successfully introduced and deployed using onlay fusion guidance. Sequential stenting of all 4 fenestrations with balloon-expandable covered stents was completed without difficulty. The repair was extended distally using a bifurcated stent-graft and bilateral iliac limb extensions. Final rotational DSA and CBCT revealed widely patent CA, SMA, and bilateral renal arteries with no evidence of type I or III endoleak (Figure 3). The patient developed bilateral lower extremities weakness, which resolved after the placement of cerebrospinal fluid drainage. The patient was dismissed on a postoperative day 13 with no other complications. The follow-up CTA at 26 months revealed widely patent fenestrated-branched stent-graft but right type Ib endoleak, requiring an extension of the repair.

### Case 3

**Figure 4.**
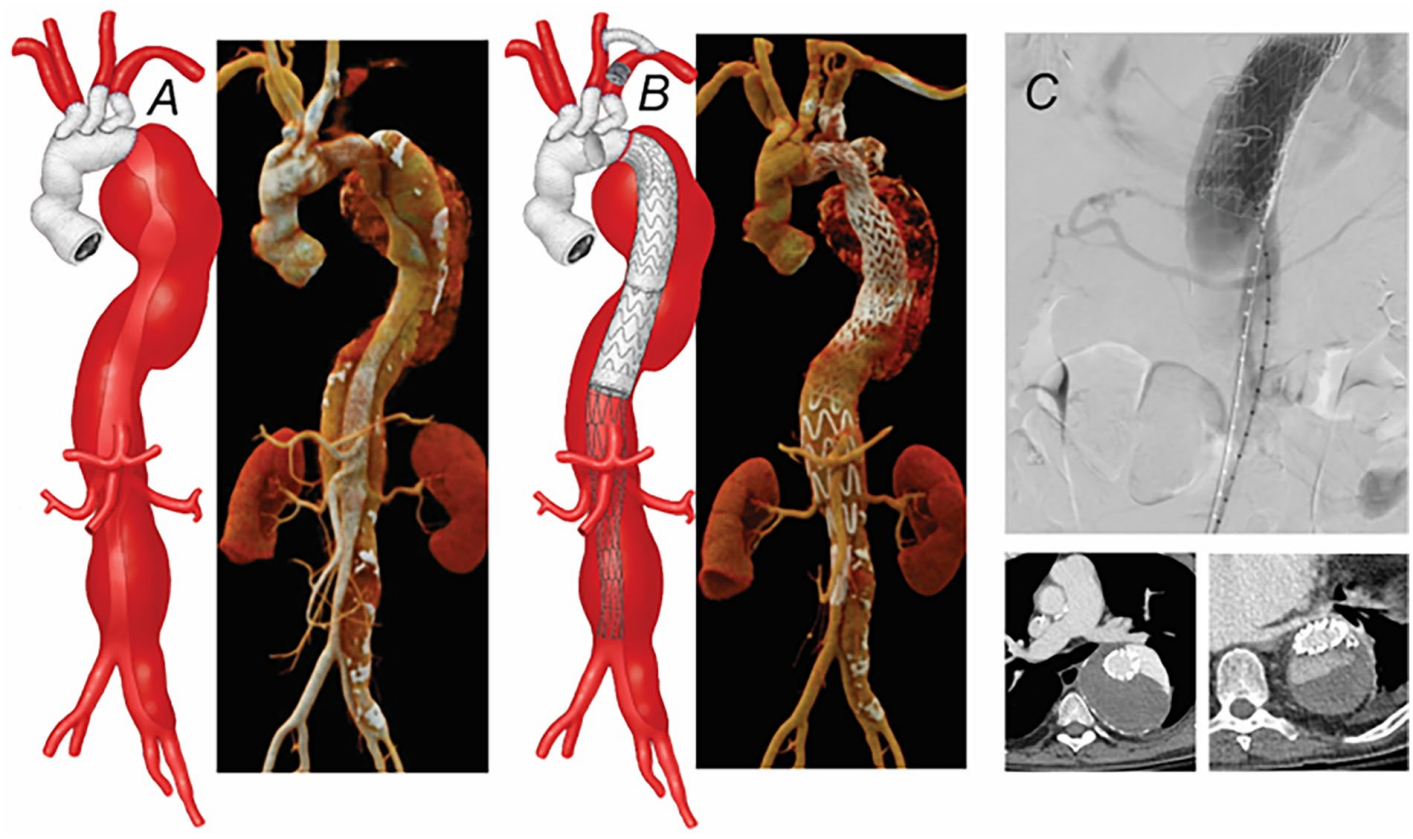
(A) Preoperative 3-dimensional computed tomography angiography (CTA) of 8 cm extent II thoracoabdominal aortic aneurysm after prior total open surgical aortic arch repair using elephant trunk technique. (B) A left carotid-subclavian bypass and thoracic endovascular aortic repair were done as a first-stage procedure. (C) After placement of the thoracic stent-graft to the level of the celiac axis, there was a stent-induced new entry tear from total disruption of the chronic dissection flap, which was treated by PETTICOAT. In-hospital repeat CTA showed a new false lumen with dense contrast opacification.

A 52-year-old male patient with Loeys-Dietz syndrome and prior total open surgical aortic arch repair using the elephant trunk technique was referred 28 months after the arch repair for endovascular repair of an enlarging 8 cm extent II TAAA. The patient had multiple complications from the prior open surgical aortic repair, including temporary dialysis, left above-knee amputation, and multiple cardiac arrests. A staged procedure was recommended, first with left carotid-subclavian bypass and TEVAR, followed by completion of FB-EVAR using off-the-shelf multi-branched stent-graft. Following placement of the thoracic stent-graft to the level of the CA, there was immediate SINE from total disruption of the chronic dissection flap despite no balloon dilatation. The false lumen was pressurized on completion angiography with poor opacification of the true lumen. To prevent the total collapse of the true lumen, PETTICOAT was performed allowing preserved expansion of the true lumen (Figure 4). The patient recovered without complications, but a pre-dismissal CTA obtained the day after the TEVAR procedure showed the development of a new dissection channel with significant contrast opacification. Urgent endovascular repair using an off-the-shelf multi-branched stent-graft was recommended.

**Figure 5.**
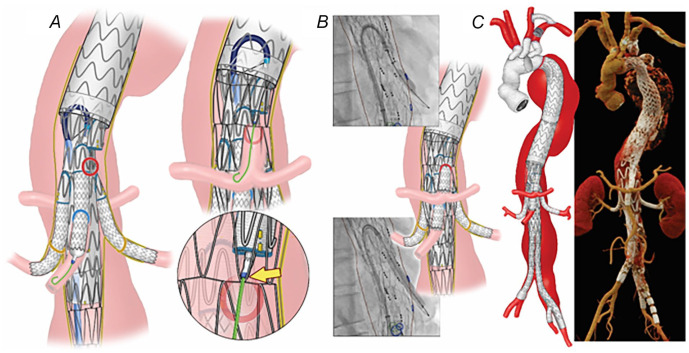
(A) Endovascular repair was performed using off-the-shelf multi-branched stent-graft through a total trans-femoral approach using a steerable sheath. Following sequential catheterization and stenting of both renal arteries and superior mesenteric artery, advancement of the stent into the celiac axis was not possible due to encroachment of the tip of the stent delivery system into one of the bare-metal stent struts. (B) A 6 Fr Shuttle sheath was introduced through the bare-metal stent struts and a short 6 × 22 mm iCAST balloon-expandable covered stent was placed. Subsequently, the celiac axis bridging balloon-expandable covered stent was successfully advanced and deployed without difficulty. (C) Postoperative 3-dimensional computed tomography angiography reconstruction shows widely patent branched stent-graft.

The second-stage branched endovascular repair was performed using a total bilateral femoral approach without brachial access. Initial access was obtained using the technique previously described with the advancement of a pigtail catheter for placement of the aortic wire. Access to the central lumen of the bare-metal stent was confirmed with intravascular ultrasound, followed by advancement of a 22 Fr 65 cm sheath and under-inflation of a compliant balloon. Once we confirmed access into the central lumen of the bare-metal stent and the onlay fusion was calibrated by pre-catheterization of one of the renal arteries, the off-the-shelf multi-branched stent-graft was oriented extra-corporeally, advanced, and deployed with each of the directional branches approximately 2 cm proximal to the intended target vessel. The repair was then extended distally into the right iliac system using universal bifurcated stent-graft and ipsilateral limb extension. The 22 Fr sheath delivery system was exchanged for a 12 Fr 33 cm Dry-Seal sheath. A Destino Bi-directional Steerable 8.5 Fr 55 cm sheath (Oscor Inc., FL) was prepared with a locking 0.014 wire introduced through the valve and looped outside. The 8.5 Fr steerable sheath was introduced into the 12 Fr sheath using a co-axial 6 Fr 90 cm Shuttle sheath instead of the original dilator. Then it was deflected by pulling the 2 ends of the pre-looped guidewire and fixed in the preferred angulation toward the target branches in the thoracic aorta. Sequential catheterization of both renal arteries and SMA, followed by stenting using a balloon-expandable covered stent was performed without difficulty. However, the advancement of the stent into the CA was not possible due to the encroachment of the tip of the stent into one of the bare-metal stent struts. Following redo catheterization and another failed attempt, we were able to introduce a 6 Fr Shuttle sheath through the bare-metal stent struts and place a short 6 × 22 mm iCAST balloon-expandable covered stent (Atrium Medical Corporation, Hudson, NH). This allowed the successful advancement and deployment of the CA bridging VBX balloon-expandable covered stent (W. L. Gore & Associates, Inc., Flagstaff, AZ). The procedure was completed with the placement of the contralateral iliac limb extension, rotational DSA, and CBCT (Figure 5). The patient required early revision of the left femoral access site due to focal dissection and was dismissed home on postoperative day 12 without other complications. Surveillance CTA at 12 months showed no evidence of type I or III endoleak, persistent type II endoleak with decreasing 7 cm TAAA aneurysm.

## Discussion

This small series shows successful FB-EVAR of chronic post-dissection TAAAs in select patients treated by previous TEVAR and PETTICOAT for TBADs or SINE. Despite the successful conduct of the procedure, technical difficulty was encountered in all 3 patients due to conflict induced by the previous bare-metal stent. The main problem to be recognized is the placement of the aortic wire through one of the struts of the aortic device. If this is not properly identified, deployment of the fenestrated-branched stent-graft in-between stent struts may be disastrous due to incomplete expansion of the device or inability to remove the delivery system. The second, less frequent difficulty, was the encroachment of catheters or the bridging stent delivery system into one of the stent struts, akin to what occurs with supra-renal fixation fenestrated-branched devices, which required placement of a smaller profile stent to allow advancement of the longer balloon-expandable covered stent.

PETTICOAT with or without stent-assisted balloon-induced intimal disruption and relamination in aortic dissection repair (STABILIZE) are known endovascular techniques for the management of acute or subacute TBADs, particularly after primary intimal tear coverage with TEVAR.^4–7^ These bare-metal stents encourage true lumen expansion and false lumen thrombosis, but should be avoided whenever possible in patients with chronic dissections who may need future FB-EVAR. Despite careful indication, some patients with PETTICOAT progress to TAAA degeneration. Canaud et al^8^ reported a systematic review of the PETTICOAT technique and demonstrated a 9% failure rate of combined proximal stent-grafting with distal bare stenting for the management of acute and chronic aortic dissection among 108 patients. In another clinical trial, Lombardi et al^9^ observed a 31% rate of complete thrombosis of the thoracic false lumen at 1-year follow-up among 40 patients with complicated acute or subacute TBAD treated with an endovascular proximal TX2 thoracic stent-graft and distal bare-metal Zenith Dissection Endovascular Stent. Although this technique is designed for the acute or subacute phase, PETTICOAT may be a bailout for SINE in select cases of chronic dissections, such as the patient with Loeys-Dietz Syndrome herein described. Although the primary intention of PETTICOAT is to expand the true lumen in acute or subacute cases, it may also be applied in patients with chronic dissection, who develop SINE, to avoid the total collapse of the true lumen due to excessive pressurization of the false lumen. The STABLE study, which included only patients with acute or subacute dissections, reported no evidence of SINE.^9^ There are other suggested methods to prevent distal SINE. Ouchi et al^10^ recommended using a stent-graft with a smaller diameter distally, followed by a standard-size proximal stent. Kolbel et al^11^ suggested the removal of the distal stent leaving the graft unsupported, akin to an elephant trunk to reduce the distal radial force. However, the aforementioned techniques have been infrequently used and have not been compared with PETTICOAT.

The feasibility of FB-EVAR following PETTICOAT has not been previously reported in the literature. The concern with subsequent FB-EVAR derives from the experience of endovascular repair in patients with stents using supra-renal fixation devices or the Endologix Powerlink XL (Endologix, Inc., Irvine, CA) stent-graft, which has the stent structure inside the device lumen, predisposing to wire entrapment in and out of stent struts. Catheterization of the renal and mesenteric vessels may also be technically difficult if the strut is obliterating the origin of the vessel. Stent-graft design is similar to what is used for other patients with chronic dissections and relies on inner aortic diameter and vessel orientations, with a preference for directional branches for vessels originating from wider aortic diameter and with down-going orientation. In terms of bridging stent selection, our preference is to use iCAST covered stents for fenestration and a balloon-expandable stent for directional branches due to its lower profile and higher radial force, which is needed to offset the risk of compression from the PETTICOAT.

A few maneuvers need to be highlighted to prevent or overcome these complications. First, advancement of the guidewire may require the use of a pigtail catheter to cross the segment that has bare-metal aortic stents. Once this is done, it is important to confirm adequate wire placement using intravascular ultrasound or a partially inflated compliable balloon, which is retracted in a similar fashion as a Fogarty balloon catheter with careful attention to avoid stent dislodgment. We also found that the advancement of a long 20 to 22 Fr sheath is very useful as a final third maneuver. If the wire is placed behind one of the stent struts, we recommend keeping the large sheath at that level and using a “buddy” pigtail catheter to advance a new wire from that level upward. The maneuver is repeated until one confirms adequate access. Finally, vessel catheterization may require a similar maneuver or placement of a smaller profile stent to shift the bare-metal strut and allow advancement of the intended bridging covered stent as described in patient 3.
